# Super-resolution images of peptidoglycan remodelling enzymes at the division site of *Escherichia coli*

**DOI:** 10.1007/s00294-018-0869-x

**Published:** 2018-07-28

**Authors:** Bill Söderström, Helena Chan, Daniel O. Daley

**Affiliations:** 1 Structural Cellular Biology Unit, Okinawa Institute of Science and Technology, Onna, 904-0495 Japan; 2 Department of Biochemistry and Biophysics, Stockholm University, Stockholm, 106 91 Sweden

**Keywords:** *E. coli*, Cell division, FtsZ, FtsI, FtsN, Peptidoglycan

## Abstract

Bacterial cells need to divide. This process requires more than 30 different proteins, which gather at the division site. It is widely assumed that these proteins assemble into a macromolecular complex (the divisome), but capturing the molecular layout of this complex has proven elusive. Super-resolution microscopy can provide spatial information, down to a few tens of nanometers, about how the division proteins assemble into complexes and how their activities are co-ordinated. Herein we provide insight into recent work from our laboratories, where we used super-resolution gSTED nanoscopy to explore the molecular organization of FtsZ, FtsI and FtsN. The resulting images show that all three proteins form discrete densities organised in patchy pseudo-rings at the division site. Significantly, two-colour imaging highlighted a radial separation between FtsZ and FtsN, indicating that there is more than one type of macromolecular complex operating during division. These data provide a first glimpse into the spatial organisation of PG-synthesising enzymes during division in Gram-negative bacteria.

## Introduction

Cell division in bacteria requires more than 30 different proteins (de Boer 2010). These proteins act in a co-ordinated manner to bring about chromosome segregation, constriction and peptidoglycan (PG) remodelling. How the biochemical activities of these proteins are co-ordinated remains an unsolved mystery. In large part, this can be attributed to the fact that we do not fully understand how division proteins interact in time and space. Structural information on the organisation of proteins at the division site has therefore been a long-standing, yet elusive goal of the cell division community. Unfortunately, owing to the transient nature of this protein assembly little progress has been made by traditional biochemical and structural approaches. However super-resolution fluorescence imaging has emerged as a powerful method for gaining structural insight into the division machinery (Holden 2018). It reveals information about the spatial organisation of proteins in vivo, and to some extent, their dynamics and interacting partners (if two proteins are imaged simultaneously).

A number of studies have utilised super-resolution fluorescence imaging to determine the spatial organisation FtsZ at midcell. FtsZ is considered a key player in division as it is the first protein to arrive at the division site and acts as a recruitment base for other proteins, such as those required for peptidoglycan (PG) remodelling. At the division site it forms proto-filaments that are anchored to the membrane by FtsA and ZipA, and once membrane anchored, these filaments can exert a constrictive force (Osawa et al. 2008, 2009). Initial super-resolution imaging revealed that FtsZ, FtsA and ZipA form patchy rings (or toroids) at the division site (reviewed in Haeusser and Margolin 2016; Holden 2018). Structured illumination microscopy (SIM) imaging suggests that the patchy rings formed by FtsZ, FtsA and ZipA constrict simultaneously, but that proteins involved in PG remodelling are not in the patchy rings (Söderström and Daley 2016; Söderström et al. 2016). However, only a limited amount of information can be gathered from this type of super-resolution imaging, as the resolving power of SIM does not generally exceed 120 nm.

**Fig. 1:**
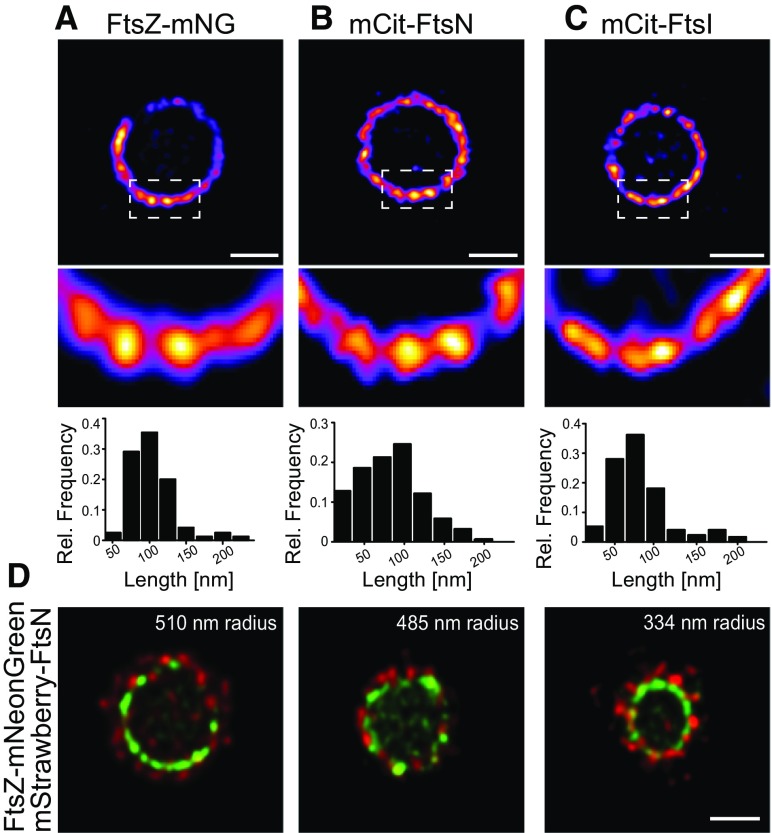
Super-resolution gSTED images of divisome proteins. **a** FtsZ–mNeonGreen (mNG). Average density dimensions; length = 109 nm, thickness = 80 nm. **b** mCitrine (mCit)-FtsN. Average density dimensions; length = 106 nm, thickness = 101 nm. **c** mCitrine (mCit)-FtsI. Average density dimensions; length = 96 nm, thickness = 77 nm. **d** FtsZ–mNGreen and mStrawberry–FtsN. All images were obtained by time-gated stimulated emission depletion (gSTED) nanoscopy of *E. coli* cells immobilised in a vertical position using micron holes produced in agarose pads. Scale bars 500 nm. Data on FtsZ and FtsN have been published previously (Söderström et al. 2018) and are reprinted with permission from Wiley and sons. mCitrine–FtsI was expressed in strain MG1655 from the *pRha*67 plasmid using 2 mM rhamnose. Technical details on micron hole preparation and imaging are presented in (Söderström et al. 2018)

Bisson-Filho et al. (2017) have developed an approach to trap and image *Bacillus subtilis* cells in a ‘standing’ (i.e., vertical) position using agarose pads with arrays of micron sized holes. Using this simple change of imaging perspective, it was possible to image proteins around the division site without the need for 3D image reconstruction. In a recent study we used agarose pads to trap *E. coli* cells and image division proteins using time-gated stimulated emission depletion (gSTED) nanoscopy, which has a resolution well below 60 nm (Söderström et al. 2018). When we imaged the native FtsZ and an FtsZ–mNeonGreen fusion, we observed discrete densities, that were distributed as a pseudo-ring around the division site (Fig. 1a) (Söderström et al. 2018). The dimensions (and sometimes the orientation) of the densities varied, but the images suggest that they were on average ~ 110 nm long and ~ 70 to 80 nm thick and covered approximately 65% of the division site.

The FtsZ densities are known to be dynamic, ‘treadmilling’ around the division site (Bisson-Filho et al. 2017; Yang et al. 2017). In doing so they govern the localisation of PG remodelling enzymes, thus coupling FtsZ constriction to PG remodelling. We were further motivated to understand how PG remodelling proteins are spatially organised at the division site, as we believed it would give clarity into the coupling between FtsZ and the PG remodelling enzymes. Initially we focused our attention towards the FtsN protein, an allosteric activator of PG remodelling proteins that initiates envelope constriction (Weiss 2015). In our experiments, *E. coli* cells were vertically immobilised and gSTED nanoscopy was again used to image the native FtsN, as well as an mCitrine–FtsN fusion (Söderström et al. 2018). The resulting images initially resembled those of FtsZ; they indicated that FtsN formed a pseudo-ring with discrete densities that were distributed around the division site. The densities were on average 110 nm in length and 100 nm in thickness, and they were also dynamic. However, in contrast to FtsZ, the FtsN densities covered a larger average percentage of the pseudo-ring, approximately 80% (Fig. 1b). Although not presented in the original paper we have also probed the distribution of the PG transpeptidase FtsI (also as an mCitrine fusion). Again, we observed a pseudo-ring with dynamic densities that were distributed around the division site. The densities were on average 96 nm in length, 77 nm in thickness and covered roughly 75% of the septal ring (Fig. 1c).

What do the densities seen by super-resolution microscopy represent? The dimensions of the densities for FtsZ, FtsI and FtsN are consistent with large, discrete macromolecular complexes. This observation is supported by protein: protein interaction data, which indicate that FtsZ, FtsI and FtsN physically interact with a number of other divisome proteins (Alexeeva et al. 2010; Buddelmeijer and Beckwith 2004; Di Lallo et al. 2003; Fraipont et al. 2011; Karimova et al. 2005; Müller et al. 2007; Pazos et al. 2013). It has been assumed that all division proteins are assembled into a single macromolecular complex, which is referred to as the divisome. But intriguingly, dual-colour gSTED images revealed that the densities of FtsZ and FtsN did not overlap at the division site. The spatial separation was most obvious as the cells started to constrict, as the FtsZ densities formed a pseudo-ring ‘inside’ that of the FtsN pseudo-ring (Fig. 1d) (Söderström et al. 2016, 2018). Although it has not yet been explicitly resolved whether FtsI co-localises with FtsN during division, super-resolution SIM data do indicate that the FtsI and FtsN pseudo-rings constrict simultaneously (Söderström and Daley 2016; Söderström et al. 2016).

As always, new data generates new insight, but also more unanswered questions. The latest super-resolution fluorescence images of FtsZ and the PG remodelling proteins FtsI and FtsN indicate that all exist in large dynamic macromolecular complexes. These complexes are spatially separated when visualised by super-resolution microscopy. This latter observation can be partially reconciled by bacterial two-hybrid data, which indicates that FtsZ largely interacts with proteins that arrive early at the division site, such as FtsA, ZipA, ZapA, ZapC, ZapD and FtsE. Whilst FtsI and FtsN largely interact with proteins that arrive late to the division site, such as FtsQ, FtsW, Pbp1b and each other. However, there are additional bacterial two-hybrid interactions that are hard to reconcile with the super-resolution images; for example, FtsZ/FtsI/FtsN all interact with ZapA, and FtsZ/FtsN both interact with FtsA. In the end, detailed knowledge of the composition of the complexes observed by super-resolution microscopy would provide important understanding of the functional units that are operating at the division site. This information is essential for re-interpreting old data, as well as designing reconstitution, biochemical and structural experiments that will ultimately reveal the molecular mechanisms that bring about cell division in Gram-negative bacteria.
